# Supplementary material for: Cell specific peripheral immune responses predict survival in critical COVID-19 patients
Source: Nat Commun. 2022 Feb 15;13:882. doi: 10.1038/s41467-022-28505-3 (PMC8847593; doi:10.1038/s41467-022-28505-3)
Supplement: Supplementary file 1 — Supplementary Information [file 41467_2022_28505_MOESM1_ESM.pdf]

Supplementary Figure 1

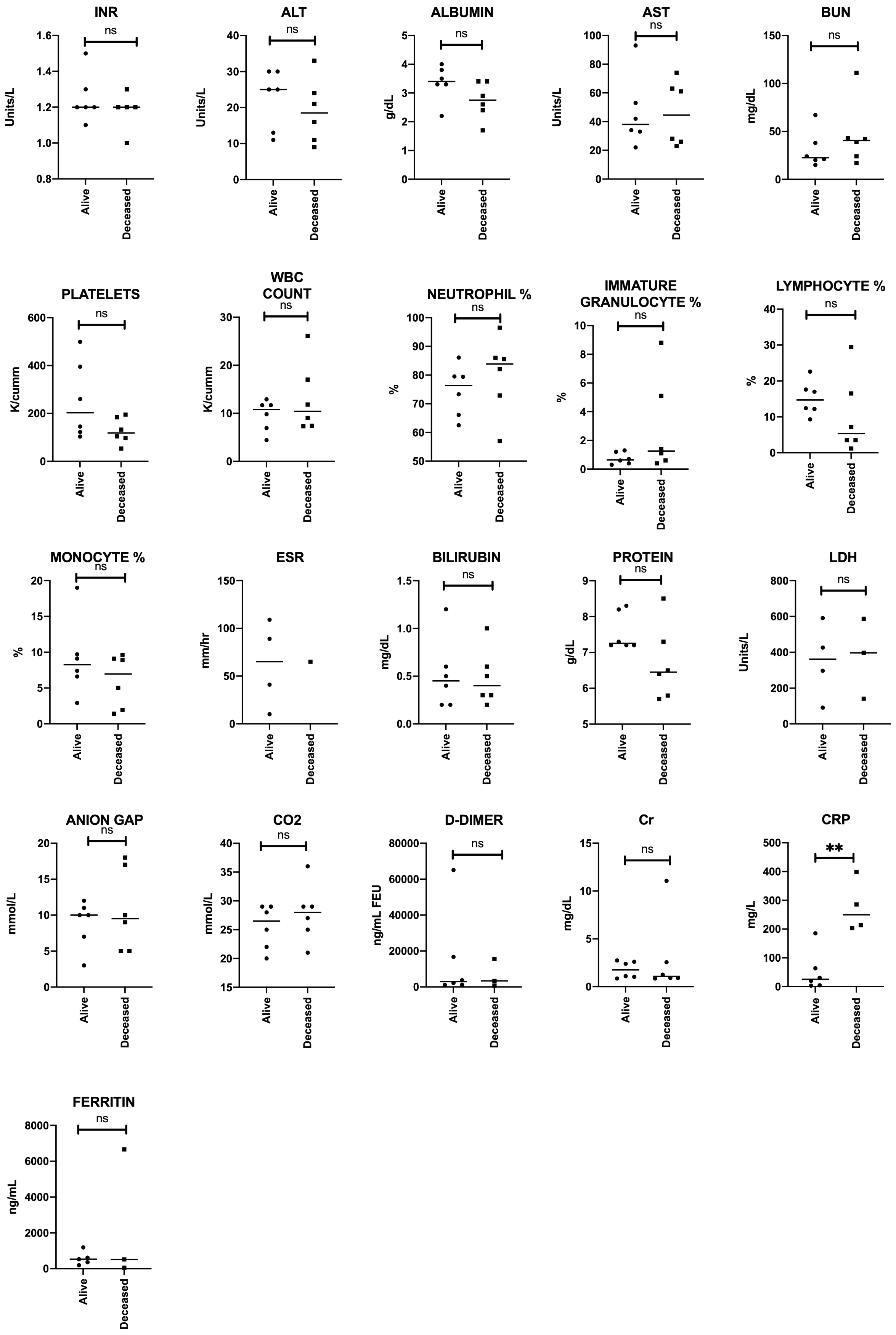

Supplementary Figure 1. Common laboratory evaluation in critical COVID-19 patients by survival outcome. Mann-Whitney statistical tests were performed. ** denotes p<0.01 and ns denotes not significant.

Supplementary Figure 2

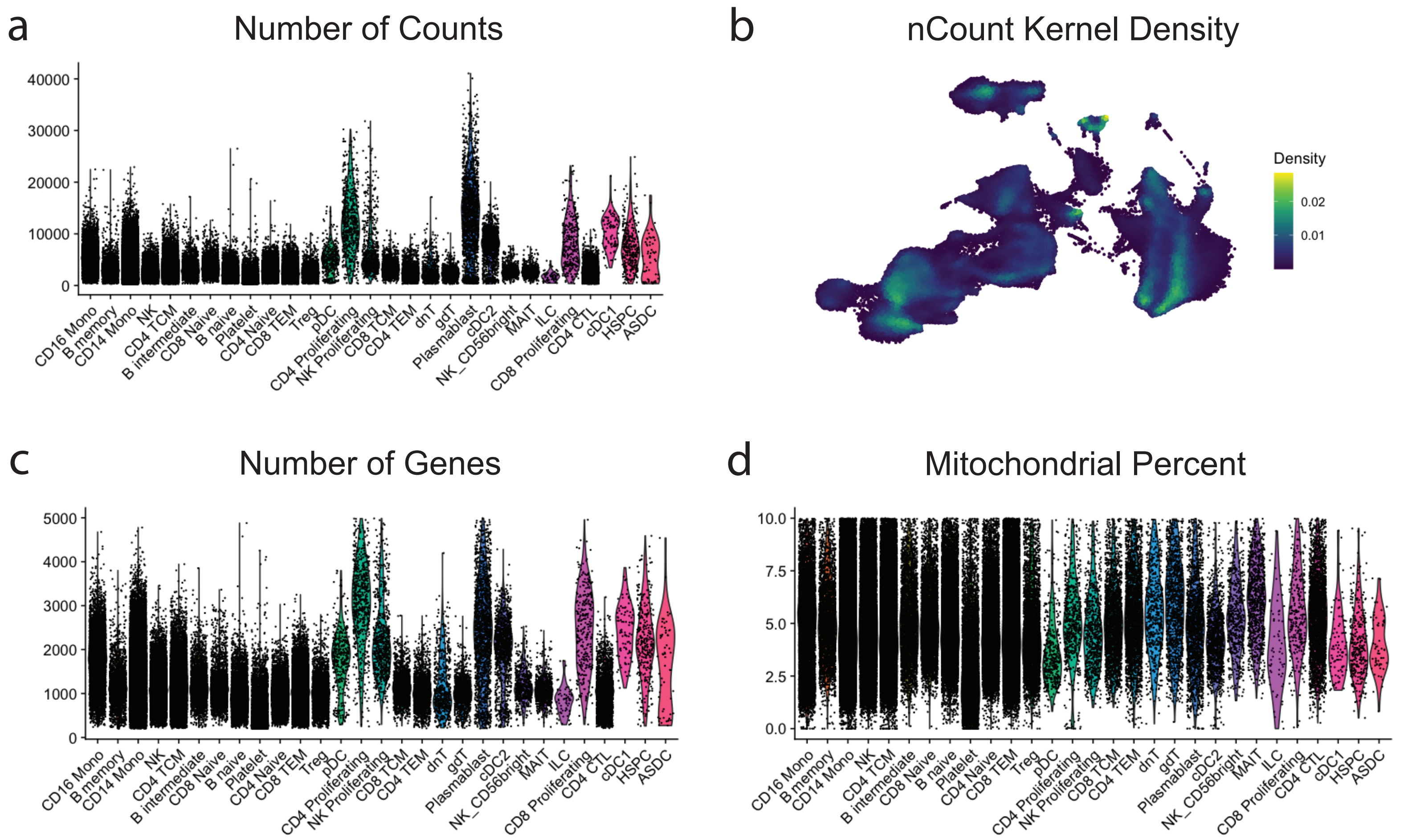

Supplementary Figure 2. scRNA-sequencing quality control metrics post-filtering by number of genes > 200 and < 5000 and percentage mitochondrial sequencing reads < 10. (a) Number of UMI counts by cell type annotation, (b) Kernel density plot using Nebulosa for (a), (c) number of genes by cell type annotation, and (d) mitochondrial sequencing read percentage by cell type annotation.

Supplementary Figure 3

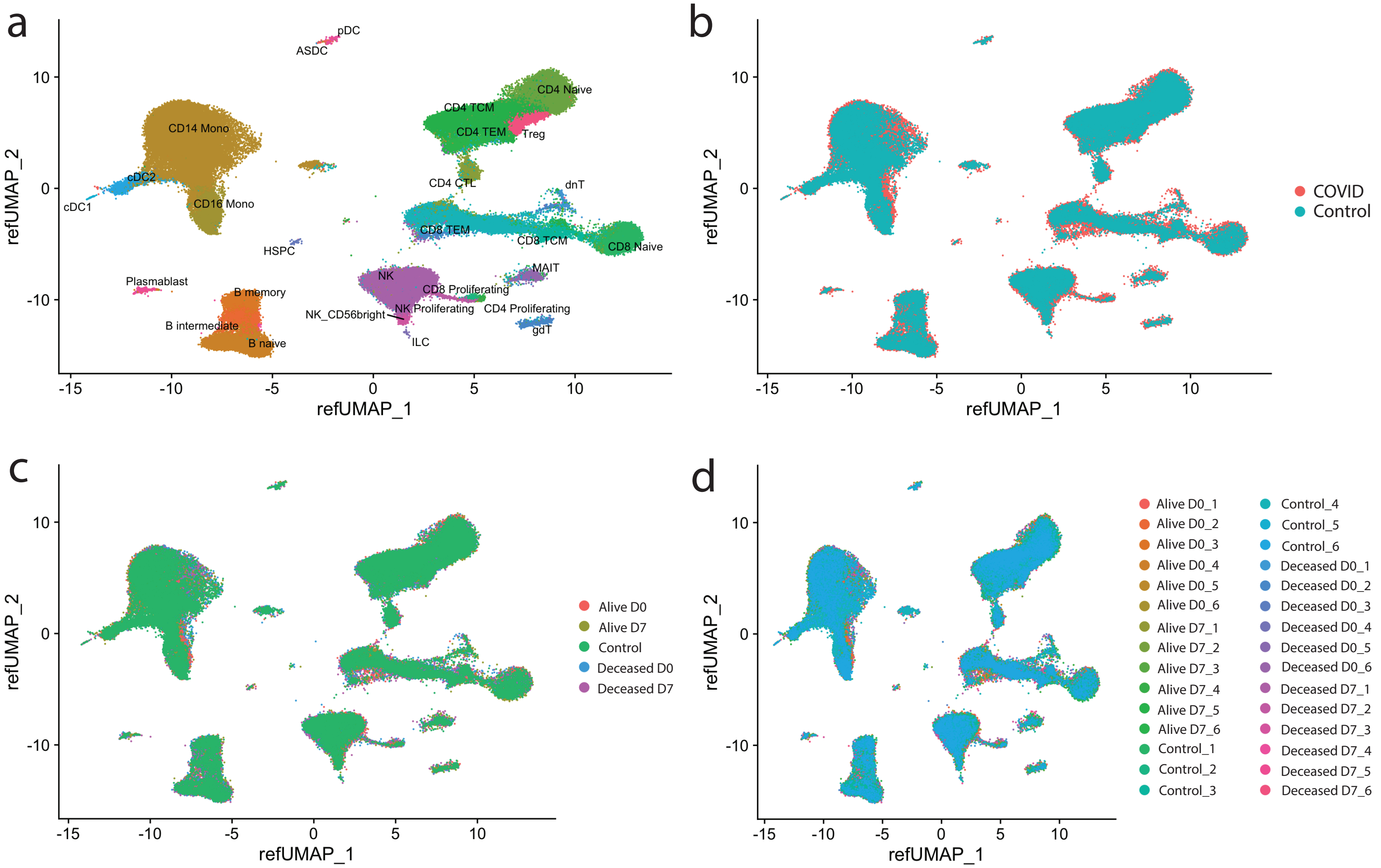

Supplementary Figure 3. Azimuth mapping to PBMC CITE-seq reference. UMAP embedding plots of PBMC scRNA sequencing profiles mapped onto a PBMC CITE-seq reference derived UMAP space via Azimuth with (a) imputed cell annotations, (b) disease status, (c) time and survival outcome, and (d) sample ID.

Supplementary Figure 4

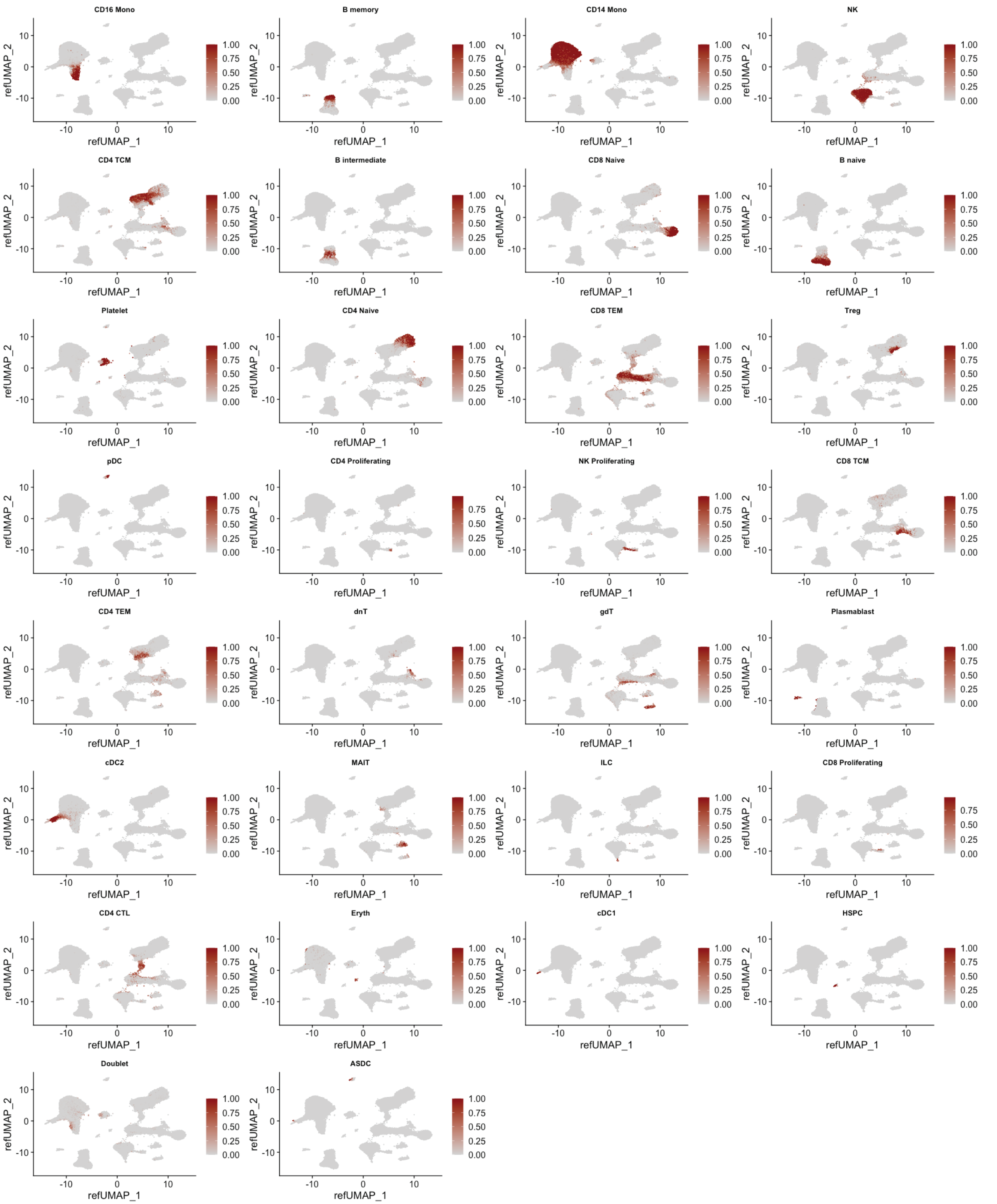

Supplementary Figure 4. UMAP embedding plots of Azimuth mapping prediction scores for all cell types annotated on the reference derived UMAP space.

Supplementary Figure 5

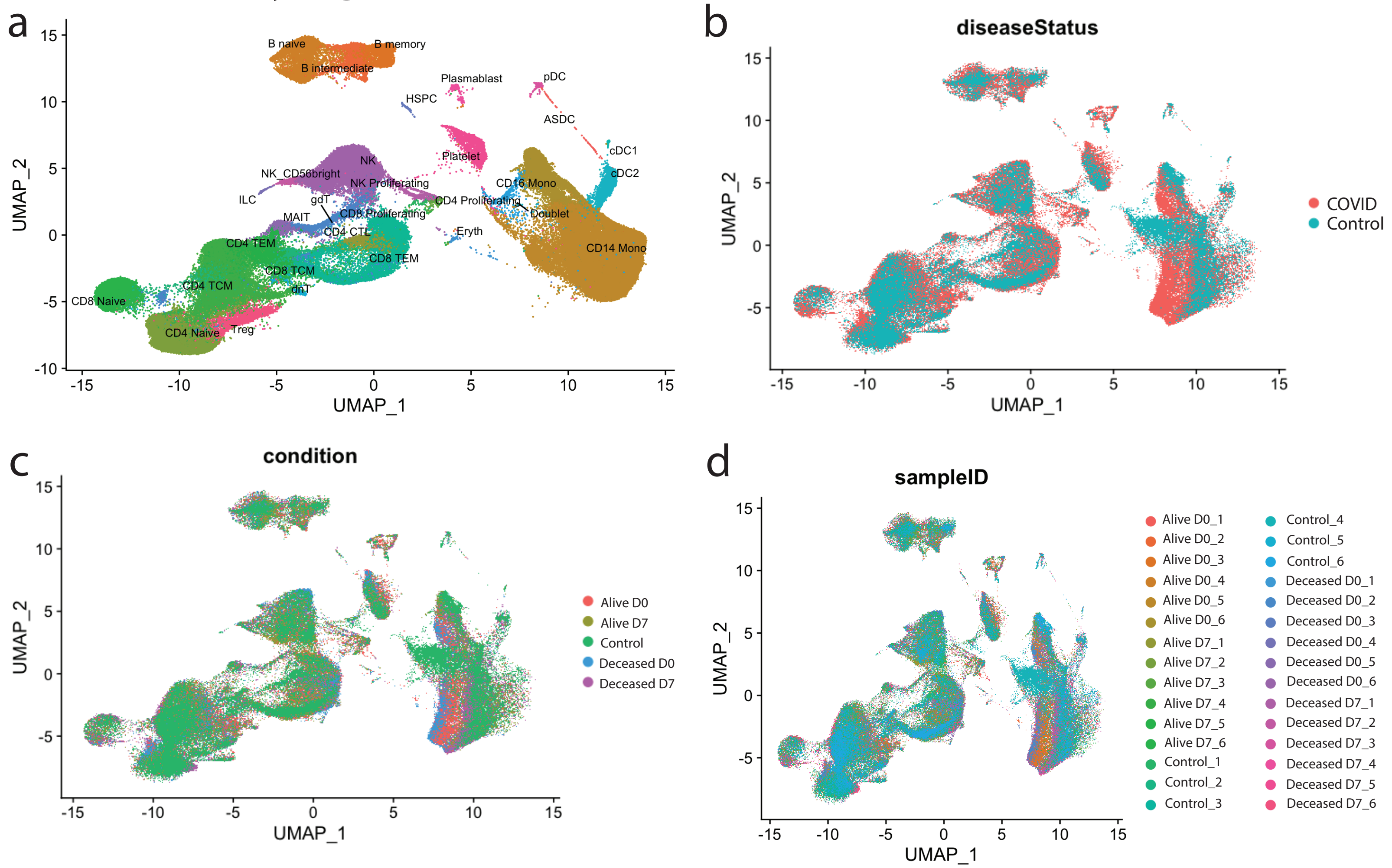

Supplementary Figure 5. Re computed UMAP with merged query and reference after Seurat multi-modal reference mapping. De novo UMAP embedding plots of PBMC scRNA sequencing profiles mapped via Azimuth with (a) reference embedding, (b) disease status, (c) time and survival outcome, and (d) sample ID.

Supplementary Figure 6

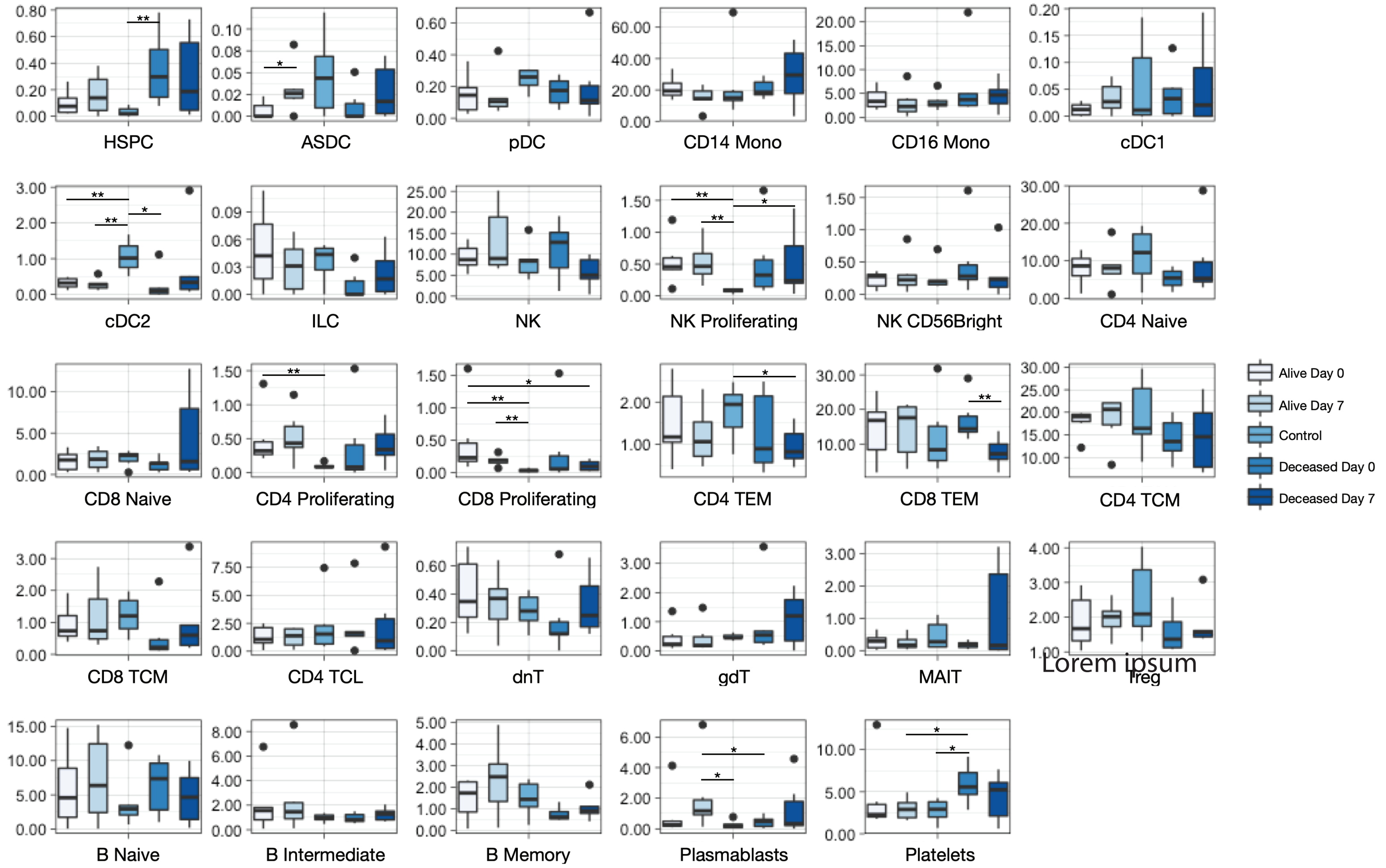

Supplementary Figure 6. Percentage of cell types annotated. Mann-Whitney statistical tests were performed. * denotes p<0.05 and ** denotes p<0.01.

Supplementary Figure 7

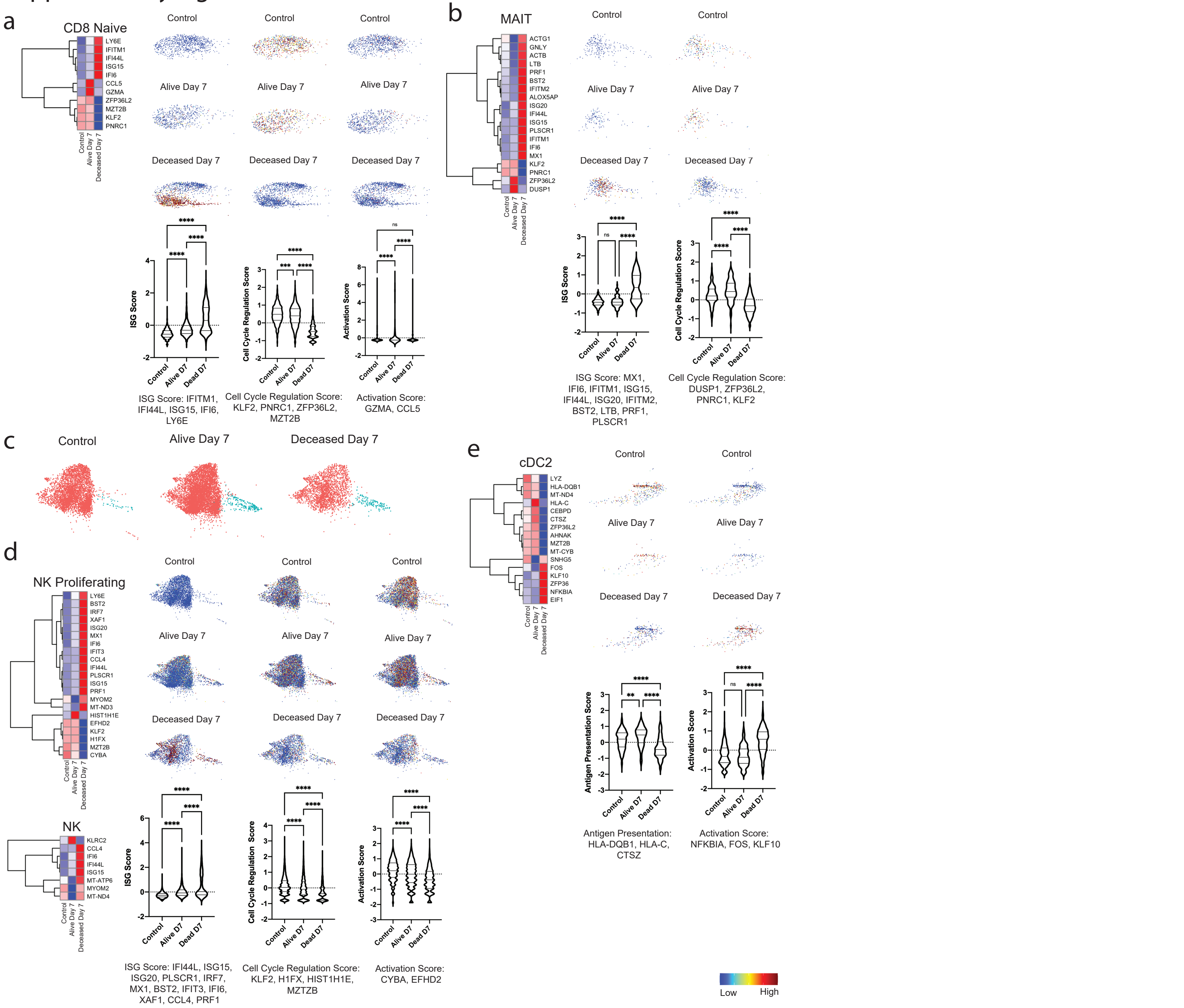

Supplementary Figure 7. Day 7 functional gene set scores calculated for Control and COVID-19 cohorts in CD8 Naïve, NK/NK Proliferating, MAIT, and cDC2 cells. (a) Hierarchical clustering heatmap (left) of average normalized gene expression for differentially expressed genes (adjusted p-value < 0.05 and log2FC > 0.50) between Control, Alive day 7, and Deceased day 7 CD8 naïve cells and their ISG z-scores, cell cycle regulation gene z-scores, and activation gene z-scores overlaid on UMAP embedding plots (right, top) with quantification (right, bottom). (b) Hierarchical clustering heatmap (left) of average normalized gene expression for differentially expressed genes (adjusted p-value < 0.05 and log2FC > 0.50) between Control, Alive day 7, and Deceased day 7 MAIT cells and their ISG z-scores and cell cycle regulation gene z-scores overlaid on UMAP embedding plots (right, top) with quantification (right, bottom). (c) UMAP embedding plots of NK and NK proliferating cells of Control, Alive day 7, and Deceased day 7. (d) Hierarchical clustering heatmap (left) of average normalized gene expression for differentially expressed genes (adjusted p-value < 0.05 and log2FC > 0.50) between Control, Alive day 7, and Deceased day 7 NK and NK proliferating cells and their ISG z-scores, cell cycle regulation gene z-scores, and activation gene z-scores overlaid on UMAP embedding plots (right, top) with quantification (right, bottom). (e) Hierarchical clustering heatmap (left) of average normalized gene expression for differentially expressed genes (adjusted p-value < 0.05 and log2FC > 0.50) between Control, Alive day 7, and Deceased day 7 cDC2 cells and their ISG z-scores and cell cycle regulation gene z-scores overlaid on UMAP embedding plots (right, top) with quantification (right, bottom). On all heatmaps blue (low) to red (high) expression. 982 Control, 787 Alive day 7, and 1,647 Deceased day 7 CD8 Naive cells were examined across 18 patients. 235 Control, 122 Alive day 7, and 397 Deceased day 7 MAIT cells were examined across 18 patients. 4,273 Control, 6,459 Alive day 7, and 2,338 Deceased day 7 NK/NK-Proliferating cells were examined across 18 patients. 553 Control, 130 Alive Day 7, and 361 Deceased day 7 cDC2 cells were examined across 18 patients. Ordinary one-way ANOVA statistical tests were used for each comparison. ** denotes p<0.01, *** denotes p<0.001, **** denotes p<0.0001, and ns denotes not significant.

Supplementary Figure 8

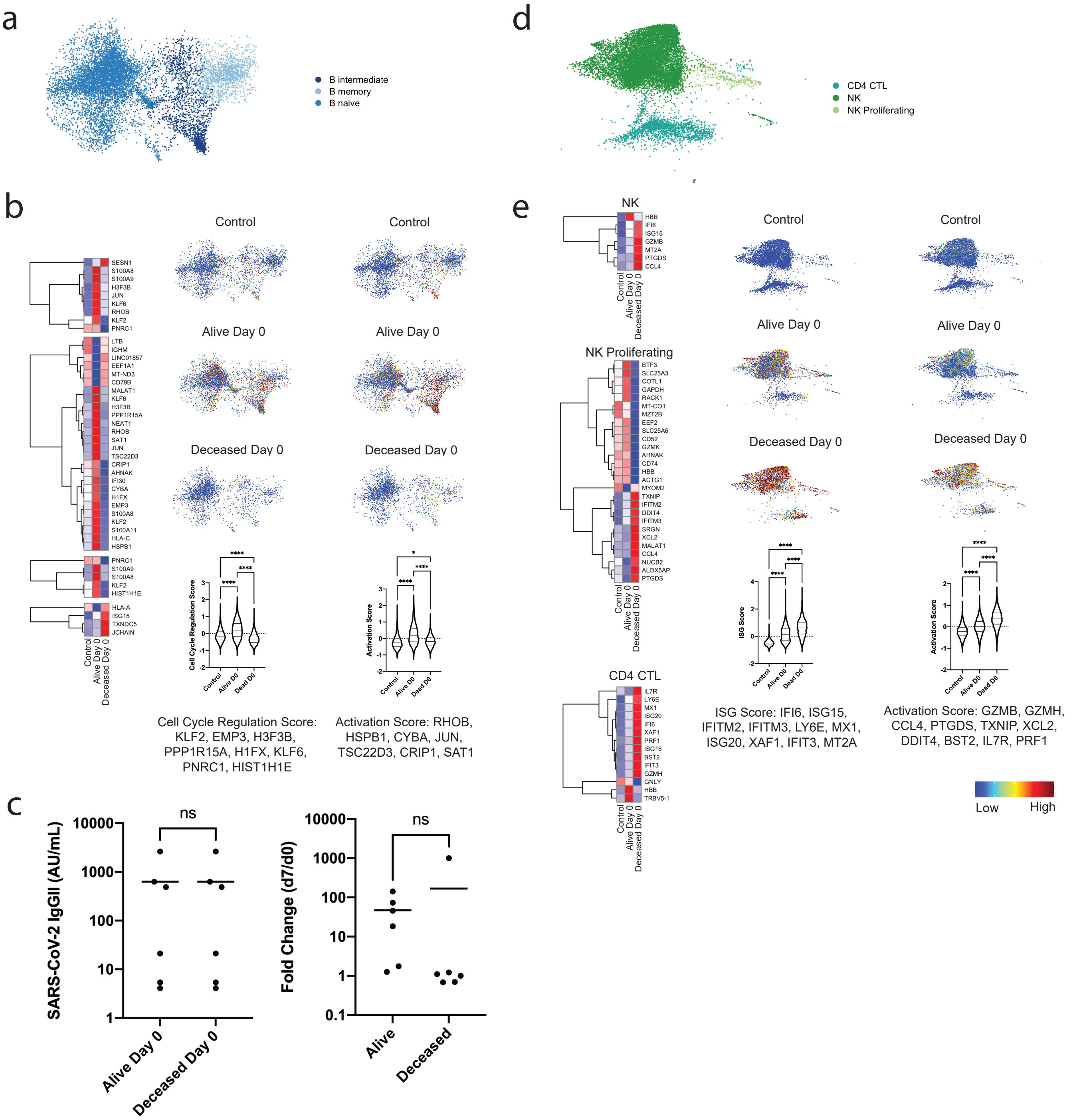

Supplementary Figure 8. Day 0 functional gene set scores calculated for Control and COVID-19 cohorts in B cell subsets, CD4 CTL, and NK/NK proliferating cells. (a) UMAP embedding plot of B-cell subsets in Control, Alive day 0 and Deceased day 0 samples. (b) Hierarchical clustering heatmap (left) of average normalized gene expression for differentially expressed genes (adjusted p-value < 0.05 and log2FC > 0.50) between Control, Alive day 0, and Deceased day 0 B-cell subsets and their cell cycle regulation gene z-scores and activation gene z-scores overlaid on UMAP embedding plots (right, top) with quantification (right, bottom). (c) SARS-CoV-2 IgG II serology in critical COVID-19 patients at day 0 split by outcome (left) and fold change (day 7/day 0) split by outcome (right). (d) UMAP embedding plot of CD4 CTL, NK, and NK proliferating cells in Control, Alive day 0 and Deceased day 0 samples. (e) Hierarchical clustering heatmap (left) of average normalized gene expression for differentially expressed genes (adjusted p-value < 0.05 and log2FC > 0.50) between Control, Alive day 0, and Deceased day 0 CD4 CTL, NK, and NK proliferating cells and their ISG z-scores and activation gene z-scores overlaid on UMAP embedding plots (right, top) with quantification (right, bottom). On all heatmaps blue (low) to red (high) expression. 3,261 Control, 4,249 Alive day 0, and 2,163 Deceased day 0 B naïve/intermediate/memory cells were examined across 18 patients. 5,511 Control, 4,082 Alive day 0, and 3,150 Deceased day 0 CD4 CTL/NK/NK Proliferating cells were examined across 18 patients. Ordinary one-way ANOVA statistical tests were used for each comparison. * denotes p<0.05, **** denotes p<0.0001, and ns denotes not significant.

Supplementary Figure 9

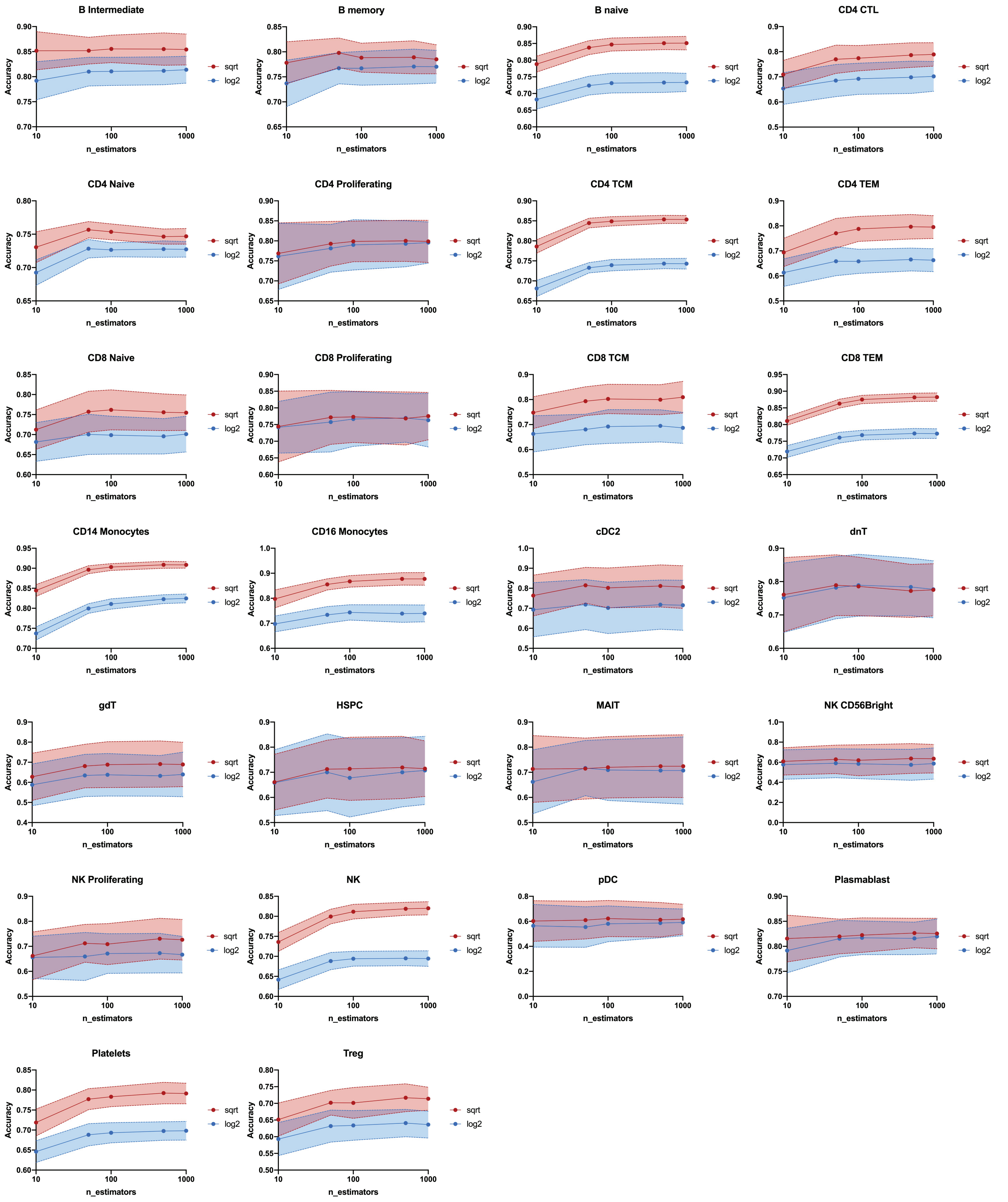

Supplementary Figure 9. Random forest classification hyperparameter tuning grid search with number of estimators and max feature (sqrt or log2) assessment using 10-fold cross validation with 5 trials (50 repeats). Mean accuracy and standard deviation plotted for each cell type annotated.

Supplementary Figure 10

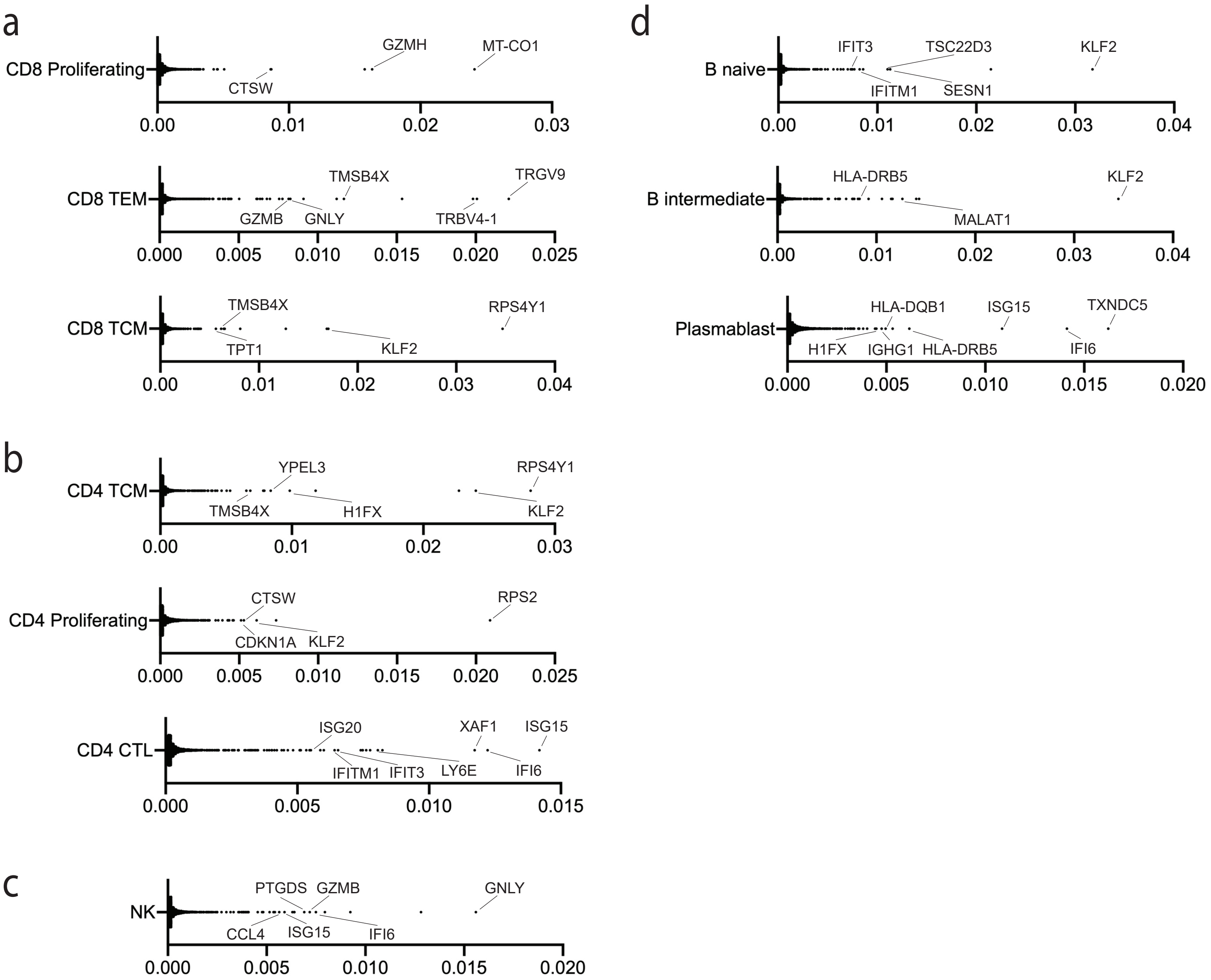

Supplementary Figure 10. Random forest classifier predicted feature importance scores calculated for key cell types. Random forest classifier ranked feature importance score for key genes annotated in (a) CD8 T-cell subsets, (b) CD4 T-cell subsets, (c) NK cells, and (d) B-cell subsets and plasmablasts.

Supplementary Figure 11

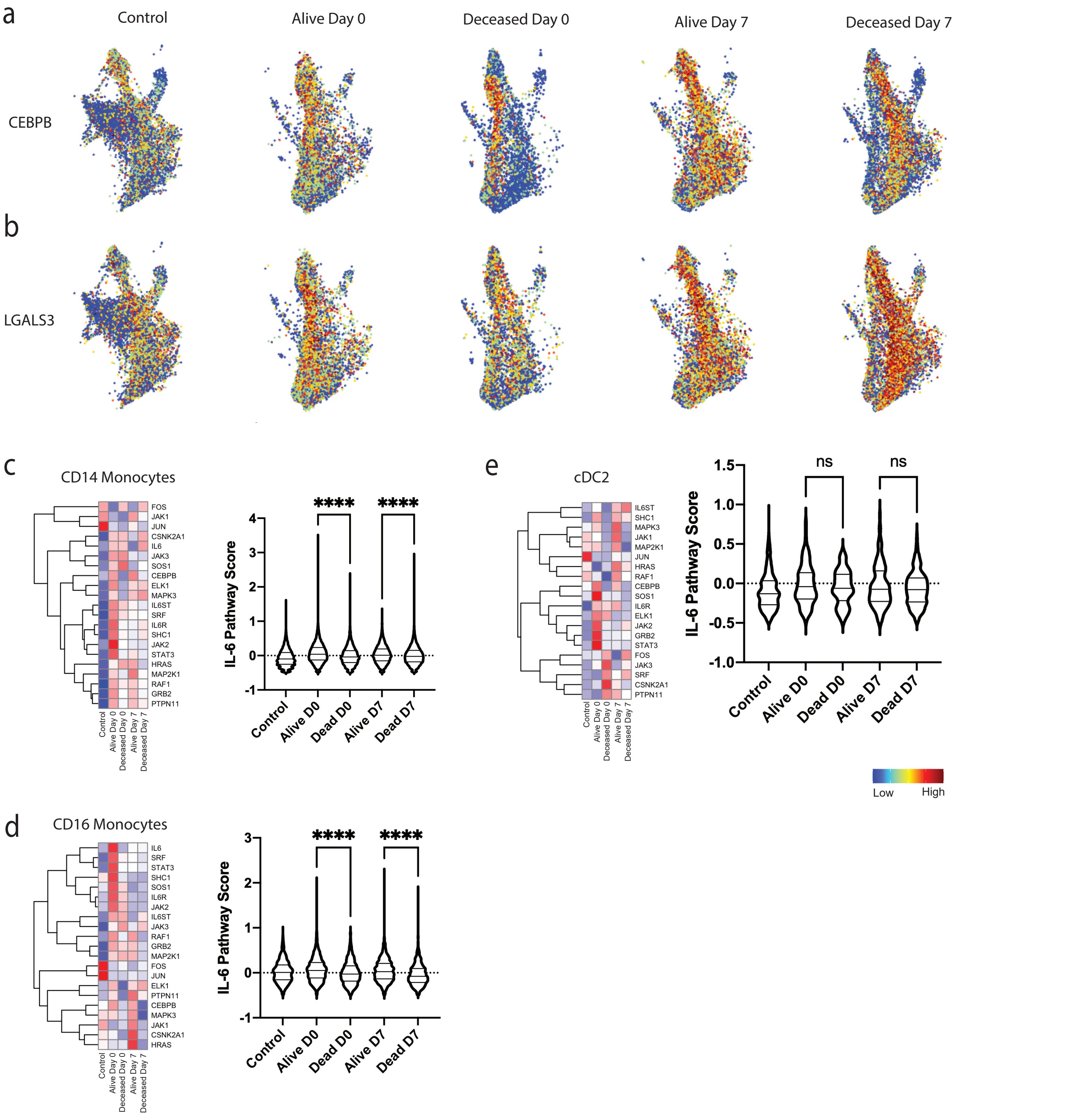

Supplementary Figure 11. IL-6 pathway enrichment in monocytes and dendritic cells. UMAP embedding plot of (a) CEBPB and (b) LGALS3 expression in CD14 monocytes, CD16 monocytes, and cDC2 cells by time and outcome. Hierarchical clustering heatmap (left) of average normalized gene expression for IL-6 signaling pathway genes and quantification (right) of IL-6 signaling pathway scores in (c) CD14 monocytes, (d) CD16 monocytes, and (e) cDC2 cells. On all heatmaps blue (low) to red (high) expression. 12,044 Control, 8,530 Alive day 0, and 5,385 Deceased day 0 CD14 Monocytes were examined across 18 patients. 1,694 Control, 1,559 Alive Day 0, and 1,216 Deceased day 0 CD16 Monocytes were examined across 18 patients. 553 Control, 108 Alive day 0, and 104 Deceased day 0 cDC2 cells were examined across 18 patients. Ordinary one-way ANOVA statistical tests were used for each comparison. **** denotes p<0.0001, and ns denotes not significant.
