# Supplementary material for: Cell specific peripheral immune responses predict survival in critical COVID-19 patients
Source: Nat Commun. 2022 Feb 15;13:882. doi: 10.1038/s41467-022-28505-3 (PMC8847593; doi:10.1038/s41467-022-28505-3)
Supplement: Supplementary file 3 — Reporting Summary [file 41467_2022_28505_MOESM3_ESM.pdf]

nature research

Corresponding author(s): Steed, Ashley

Last updated by author(s): Dec 22, 2021

Reporting Summary

Nature Research wishes to improve the reproducibility of the work that we publish. This form provides structure for consistency and transparency in reporting. For further information on Nature Research policies, see our Editorial Policies and the Editorial Policy Checklist.

Statistics

For all statistical analyses, confirm that the following items are present in the figure legend, table legend, main text, or Methods section.

n/a	Confirmed	
☐	☒	The exact sample size (n) for each experimental group/condition, given as a discrete number and unit of measurement
☐	☒	A statement on whether measurements were taken from distinct samples or whether the same sample was measured repeatedly
☐	☒	The statistical test(s) used AND whether they are one- or two-sided<br>*Only common tests should be described solely by name; describe more complex techniques in the Methods section.*
☐	☒	A description of all covariates tested
☐	☒	A description of any assumptions or corrections, such as tests of normality and adjustment for multiple comparisons
☐	☒	A full description of the statistical parameters including central tendency (e.g. means) or other basic estimates (e.g. regression coefficient) AND variation (e.g. standard deviation) or associated estimates of uncertainty (e.g. confidence intervals)
☐	☒	For null hypothesis testing, the test statistic (e.g. F, t, r) with confidence intervals, effect sizes, degrees of freedom and P value noted<br>*Give P values as exact values whenever suitable.*
☒	☐	For Bayesian analysis, information on the choice of priors and Markov chain Monte Carlo settings
☐	☒	For hierarchical and complex designs, identification of the appropriate level for tests and full reporting of outcomes
☒	☐	Estimates of effect sizes (e.g. Cohen's d, Pearson's r), indicating how they were calculated

Our web collection on statistics for biologists contains articles on many of the points above.

Software and code

Policy information about availability of computer code

Data collection	Cell Ranger version v4.0 for Barcode Identification, Alignment, Filter, Deduplication
Data analysis	Code Availability and Documentation: All scripts used for single-cell data analysis and random forest classification are available from GitHub (https://github.com/jamrute/2021_COVID_amrute_steed). [10.5281/zenodo.5748636].

Software Packages and associated packages versions:R version 4.1.1 - R environment for all single cell analysis within Seurat framework; Seurat version 4.0 - Used for single cell analysis and reference mapping; SCTransform version 0.3.2 - Used for normalization and variance stabilization; Python version 3.7.7 - Used as the environment to implement random forest classification; sci-kit learn version 0.23.2 - Used for tuning hyperparameters, cross validation, and random forest classification

Data

Policy information about availability of data

All manuscripts must include a data availability statement.

The data generated in this study have been deposited in the GEO database under accession code GSE192391.

We have no restrictions on any data or code availability.

Field-specific reporting

Please select the one below that is the best fit for your research. If you are not sure, read the appropriate sections before making your selection.

☒ Life sciences ☐ Behavioural & social sciences ☐ Ecological, evolutionary & environmental sciences

For a reference copy of the document with all sections, see nature.com/documents/nr-reporting-summary-flat.pdf

Life sciences study design

All studies must disclose on these points even when the disclosure is negative.

Sample size	We calculated that 6 samples per condition would be sufficient to detect differences at a single cell transcriptional level given that each sample yielded on average 8,000 transcriptomes.
Data exclusions	No data was excluded.
Replication	Each condition had 6 biological replicates, but each sample was sequenced once given cost constraints.
Randomization	Allocation was not random as patients were not separated into experimental groups. We did look at basic demographic variables between our groups.
Blinding	Blinding is not relevant to the study as the output parameters are not subjective and therefore not subject to this form of bias.

Reporting for specific materials, systems and methods

We require information from authors about some types of materials, experimental systems and methods used in many studies. Here, indicate whether each material, system or method listed is relevant to your study. If you are not sure if a list item applies to your research, read the appropriate section before selecting a response.

Materials & experimental systems

n/a	Involved in the study	
☒	☐	Antibodies
☒	☐	Eukaryotic cell lines
☒	☐	Palaeontology and archaeology
☒	☐	Animals and other organisms
☐	☒	Human research participants
☒	☐	Clinical data
☒	☐	Dual use research of concern

Methods

n/a	Involved in the study	
☒	☐	ChIP-seq
☒	☐	Flow cytometry
☒	☐	MRI-based neuroimaging

Human research participants

Policy information about studies involving human research participants

Population characteristics	This study utilized samples obtained from the Washington University School of Medicine's IRB approved WU350 study, a COVID-19 biorepository. Patient samples were selected based on severity of illness as defined by admission to the ICU. Those selected had available PBMC samples at day 0 and day 7 of enrollment and demographically matched. Control PBMCs were obtained from Washington University's Alzheimer's Disease Research Center specimen collection from age-matched healthy people without dementia. Average age was 70 years old and study included 11 males and 7 females.
Recruitment	Patients were recruited into Washington University's COVID biorepository study at time of hospitalization. Presentation to medical care and thus eligibility for recruitment may introduce bias.
Ethics oversight	The Washington University IRB approved the larger research study, WU350, into which these patients were recruited.

Clinical data

Policy information about clinical studies

All manuscripts should comply with the ICMJE guidelines for publication of clinical research and a completed CONSORT checklist must be included with all submissions.

Clinical trial registration	NA

Study protocol	NA
Data collection	PBMCs were collected from patient participants on day 0 and day 7 of study enrollment.
Outcomes	Patient clinical courses were followed and used to determine sample selection (mortality outcome).
